# Does Action Observation of the Whole Task Influence Mirror Neuron System and Upper Limb Muscle Activity Better Than Part Task in People With Stroke?

**DOI:** 10.1155/2024/9967369

**Published:** 2024-10-05

**Authors:** A. Sulfikar Ali, Mayur Bhat, Hari Prakash Palaniswamy, Selvam Ramachandran, Senthil D. Kumaran

**Affiliations:** ^1^Department of Physiotherapy, Manipal College of Health Professions, Manipal Academy of Higher Education, Karnataka, Manipal 576104, India; ^2^Department of Physiotherapy, Kasturba Medical College Mangalore, Manipal Academy of Higher Education, Karnataka, Manipal 576104, India; ^3^Department of Audiology and Speech Language Pathology, Kasturba Medical College Mangalore, Manipal Academy of Higher Education, Karnataka, Manipal 576104, India; ^4^Department of Speech and Hearing, Manipal College of Health Professions, Manipal Academy of Higher Education, Karnataka, Manipal 576104, India; ^5^Department of Medical Rehabilitation-Physical Therapy Program, School of Rehabilitation and Medical Sciences, College of Health Sciences, University of Nizwa, Nizwa, Oman

**Keywords:** electromyography, mirror neuron activation, movement observation, mu suppression, part task, upper extremity, whole task

## Abstract

**Background:** Task-based action observation and imitation (AOI) is a promising intervention to enhance upper limb (UL) motor function poststroke. However, whether whole/part task must be trained in the AOI therapy needs further substantiation.

**Objective:** The objective of this study is to assess and compare the mirror neuron activity and UL muscle activity during AOI of reaching task in terms of whole task (complete movement) and part task (proximal arm movements and distal arm movements).

**Methods:** In this cross-sectional study, 26 participants with first-time unilateral stroke were asked to observe the prerecorded videos of a reaching task in terms of a whole task and proximal and distal components, followed by imitation of the task, respectively. Electroencephalographic (EEG) mu rhythm suppression and electromyographic amplitude of six UL muscles were measured during the task.

**Results:** The analysis of EEG revealed a statistically significant mu suppression score, indicating mirror neuron system activity, during AOI of the whole task in C3 (*p* = <0.001) and C4 (*p* = <0.001) electrodes compared to the part task. Percentage maximum voluntary contraction amplitudes of the deltoid (*p* = 0.002), supraspinatus (*p* = <0.001), triceps brachii (*p* = 0.002), brachioradialis (*p* = 0.006), and extensor carpi radialis (*p* = <0.001) muscles showed a significant increase in muscle activity during AOI of the whole task. Also, there seems to be a task observation–specific activation of muscles following AOI of proximal or distal tasks.

**Conclusion:** The practice of the whole task should be given emphasis while framing the AOI treatment module to enhance reaching in people with stroke.

**Trial registration:** Clinical Trials Registry-India (CTRI) identifier: CTRI/2018/04/013466.

## 1. Introduction

One of the major consequences following a stroke is upper limb (UL) impairment and disability [1]. More than 60% of individuals affected with stroke have difficulty performing their activities of daily living in a normal way [2, 3]. With advances in rehabilitation, there exist several neurological treatments to enhance UL motor function such as bilateral arm training, constrained-induced movement therapy, task-oriented training, biofeedback, robotics, and virtual reality/video gaming [4]. However, these interventions are based on the execution of active movements, which is difficult for individuals with severe UL weakness during the initial phase of recovery [5]. One of the alternative treatment techniques is action observation and imitation (AOI) wherein observation and execution of an action occur sequentially [5, 6]. AOI is considered one of the rehabilitation methods for priming the motor cortex [7]. AOI consists of one person observing a motor task performed by a healthy individual, either through video [8] or through real demonstration [9]. It is based on the activation of the mirror neuron system (MNS) present in the specific areas of the brain, especially the frontal and parietal cortices [10]. Through mimicking others, AOI has been regarded as an important basis for understanding and learning an action [11, 12].

AOI is considered an effective intervention to facilitate the UL motor function [5], and it is widely incorporated reaching tasks [9, 13–16]. The type of AOI videos used in the interventions differs from simple movement [9, 16, 17], to complex functional tasks [18, 19]. Rehabilitation approaches tend to use task-oriented everyday functional movements during observation and execution, and studies have reported that task-oriented AOI is superior to simple movement–based AOI [5]. Additionally, task-oriented movement is linked to improved movement kinematics during movement execution [20]. However, if someone observes a complex whole multijoint task, e.g., writing or reaching to grasp tasks, the direct matching from the perception of the task to the execution or action of the task will be less direct, since numerous degrees of freedom are involved [21]. Also, during the early phase of stroke rehabilitation, certain movements involving isolated and/or synergistic patterns may have to be relearned. Therefore, clinicians need rehabilitation approaches that promote specific targeted motor learning without compensation [21]. As a solution, action observation of part tasks would be a better option as this practice has some degree of success, thereby motivating one to learn the skill when compared with the whole task [22]. Therefore, task-based AOI with a whole task or part task needs further substantiation. Earlier researchers also looked into the effect of AOI of the whole task and part task on improving cortical activity and muscle in normal healthy individuals [21, 23]. However, it is still unclear whether it applies to stroke rehabilitation.

AOI leads to neuroplastic changes in the brain based on the findings from functional magnetic resonance imaging (fMRI) and behavioral outcome measures in acute [17] and chronic [24] stroke subjects. However, most of the studies have used functional outcomes like Fugl-Meyer–upper extremity (FM-UE) assessment, box and block test, action research arm test, and movement time to assess the efficacy of AOI [9, 17, 18, 25], whereas only a few studies have used outcomes like fMRI [17, 24], electroencephalography (EEG) [14], and electromyography (EMG) [26], to assess mirror neuronal activation and muscle activity following AOI, which in turn gives an idea about the cortical functional connectivity and peripheral muscle activity. Suppression of mu rhythm in EEG is considered an indicator for the MNS activation centrally [27], whereas the impact of MNS activation can be assessed peripherally by measuring muscle activity using EMG [26].

Therefore, there is a need to assess the influence of the whole task and part task during the delivery of AOI on improving MNS activity and muscle activity in people with stroke. In this study, our objective was to assess MNS activation using EEG and UL muscle activity using surface EMG during AOI of reaching task in terms of the whole task (complete movement) and part task (proximal arm movements and distal arm movements).

## 2. Methods

### 2.1. Study Design and Setting

A cross-sectional design was carried out at the Neuromotor Control Unit, Department of Physiotherapy, and Department of Speech and Hearing, Kasturba Hospital, Manipal, India. Before the onset of the study, approval was sought and obtained from the institutional research and ethics committees (IEC number: 66/2018) of Kasturba Medical College and Kasturba Hospital, Manipal, India.

### 2.2. Study Participants

Twenty-six individuals aged between 18 and 80 years, of either sex, diagnosed with unilateral stroke having Brunnstrom motor recovery stage (BMRS) ≥ 2 to ≤ 6 in the affected UL and hand [28], Montreal cognitive assessment score (MOCA) ≥ 26 [29], and intact ability to imitate score ≥ 8 with nonparetic UL [30], were included in the study. Before enrollment, all included individuals gave their informed consent after receiving a detailed explanation of the study procedure.

### 2.3. Experimental Setup and Procedure

Participants were instructed to relax and be seated comfortably with their arm placed on the armrest of a chair with shoulder 10-degree abduction, elbow 90-degree flexion, forearm pronation, and wrist in neutral position. A table was placed 2 in. apart from the arms of the chair. A cylindrical shaped glass was kept on the table which was positioned one arm distance from the participants. Every participant received a brief explanation of the surface EMG and EEG procedures.

**Figure 1:**
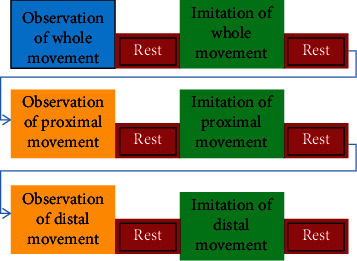
The box car paradigm consists of observation of whole, proximal, and distal movements followed by imitation of the same movements, respectively.

**Figure 2:**
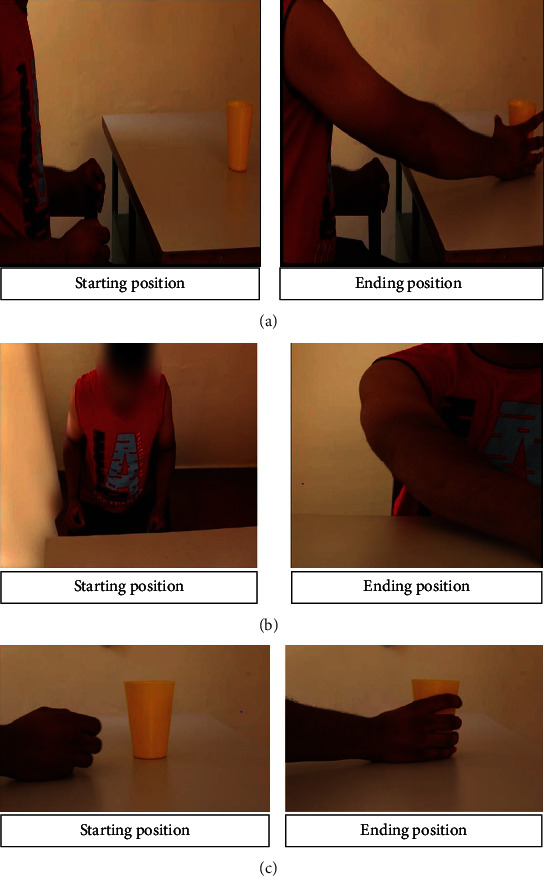
Illustration of different AOI conditions. (a) Starting and ending position of a complete reaching task performed by a normal individual without any abnormal compensatory movements. (b) Starting and ending position of a reaching task performed by the participant with proximal movement (shoulder and elbow component). (c) Starting and ending position of a reaching task performed by the participant with distal movement (wrist component).

Participants were asked to observe three prerecorded videos from their own perspective, each lasting for 2 min (showing the task repeatedly) followed by an imitation of the observed task. Stimuli were shot with a camera from superior, anterior, posterior, and lateral angles and projected on a computer screen. In this study, a series of three videos which a normal healthy individual performed were presented to participants in a sequence of ways, as represented in the form of a box car paradigm in Figure 1. Videos included (a) the whole task video, reach-to-grasp movement performed with the entire UL; (b) proximal component video, shoulder and elbow movements during reach-to-grasp task, and (c) distal component video, wrist movement during reach-to-grasp task (Figures 2(a), 2(b), and 2(c)). Participants were asked to imitate the task followed by observation of respective videos. An adequate rest period of 2 min was given after each observation and imitation to avoid the carryover effect from the active block. Verbal cues were given to the participant during the AOI of the task. The whole procedure lasted for 30 min. EEG mu rhythm suppression and normalized EMG muscle activity (percentage maximum voluntary contraction (%MVC)) were recorded during observation and imitation of the whole task and proximal and distal movement components.

### 2.4. EEG Recording and Data Processing

**Figure 3:**
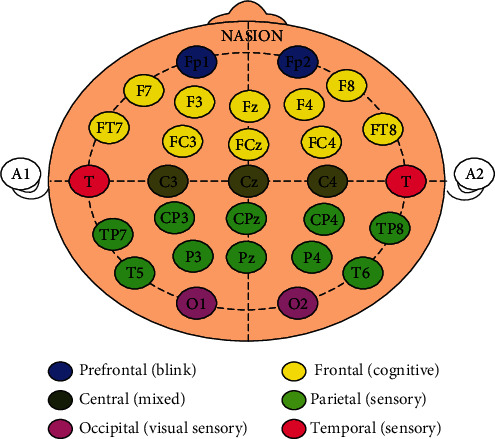
Electrode placement for electroencephalography (EEG) using a 32-channel electrode cap according to the 10-20 classification. The electrodes are color-coded, and the regions of interest are indicated.

For EEG recording, skin prepartion gel (Quick Cell and Nuprep) was used to prepare the electrode placement sites. Conduction paste (Quick Cell Electrolyte and Ten20) was applied to enhance conduction, and a 32-channel electrode cap was placed on the participant's scalp with reference electrodes on the mastoid process on both sides (Figure 3). Every electrode site's impedance was kept below 5 k*Ω*, while the interelectrode impedance was kept under 2 k*Ω*. Neuroscan 4.5 was used to acquire brain response, and EEG was recorded using Compumedics Neuroscan Systems (neuroscan laboratories, Australia).

Raw EEG data was captured with bandpass filters set between 1 and 100 Hz; sampled at 1000 Hz and analyzed using MATLAB (2017a). First, EEG channels were identified using a standard template (BESA 4 shell dip fit spherical model), and the continuous EEG data were visually inspected for irregularities due to open channels. The spherical interpolation approach was utilized in the MATLAB window's command line to interpolate participant motions. Subsequently, the data is exposed to bandpass filtering, where the cutoff frequency ranges from 1 to 30 Hz. Raw EEG files were converted to average files by epoching them with a time window of 0 to 500 ms with reference to stimulus onset. With the help of the EEG Lab's “runica” command, multivariate event-related potential (ERP) waveforms were broken down into their subcomponents according to their source using independent component analysis (ICA). The ICA-processed waveforms were then analyzed using multiple artifact rejection algorithms (MARA) [31], which automatically eliminates the components containing artifacts depending on several factors, followed by waveforms which was referred to as common average. Re-referencing and averaging were done using a MATLAB code. Absolute power for mu frequency bands in millivolts (mv) was derived for two central electrodes (C3 and C4) overlying the sensorimotor cortex. We selected sites C3 and C4 near the sensory-motor area, which are used to record the mu rhythm activity [27], and widely reported in the action observation studies [14, 32, 33].

### 2.5. EMG Recording and Data Processing

Delsys Trigno Wireless EMG System (8 channel) (AD instruments, United States; model number-DSY-DS-T01D-4, 2016) was utilized to capture muscle activity from six muscles of affected UL which include anterior deltoid, supraspinatus, biceps brachii, long head of triceps brachii, brachioradialis, and extensor carpi radialis (ECR) [34]. Out of these six muscles, anterior deltoid, supraspinatus, biceps brachii, and triceps brachii are considered proximal muscles whereas brachioradialis and ECR are considered as a distal muscle. With the participant's permission, the exposed body portion was cleansed with sterillium (propanol) to lessen skin resistance. Wireless EMG sensors were placed on the skin over the muscle bellies in parallel according to the guidelines of Surface Electromyography for the Non-Invasive Assessment of Muscles (SENIAM) recommendations [35]. The sensors were attached to the skin by using adhesive tape. EMG signals were recorded at 1000 Hz and preamplified (0–1.5 mv) using a Delsys electromyography system. The LabChart software (version 8.1.13, LabChart, AD Instruments, Australia, model number-MLS060/8) was used to amplify the wireless EMG signals (×1000), bandpass filter them (20–500 Hz), and sample them at 4 kHz and then processed with root mean square (RMS) using 20-ms epochs. RMS mean amplitude was calculated, and data entry was done. EMG amplitudes obtained while imitating the movements were normalized and expressed as %MVC. %MVC was calculated using the formula (mean amplitude/maximum amplitude) × 100 [36].

### 2.6. Statistical Analysis

Data analysis was done using the statistical package for the social sciences version 16.0 (SPSS Inc. (2007), Chicago (III), United States). The Shapiro-Wilk test was used to check the normality of the data. Since the data was skewed, Friedman's ANOVA was used to analyze EEG data of the C3 and C4 electrodes and %MVC EMG amplitudes across AOI of whole, proximal, and distal tasks. A 2 × 3 Friedman's ANOVA was conducted to analyze the EEG amplitude for three tasks, normal whole movement, proximal movement (part task), and distal movement (part task), during two conditions (observation and imitation) of the task separately for C3 and C4 electrodes. A 3 × 6 Friedman's ANOVA was performed to analyze the EMG amplitude for six muscles (anterior deltoid, supraspinatus, biceps brachii, triceps brachii, brachioradialis, and ECR) during the three tasks (normal whole movement, proximal movement (part task), and distal movement (part task)). The post hoc analysis was performed using the Wilcoxon signed rank tests with the Bonferroni adjustments to analyze the mu suppression score and %MVC EMG amplitudes between the test conditions to identify which group differed from each other.

## 3. Results

### 3.1. General Characteristics of the Participants

**Figure 4:**
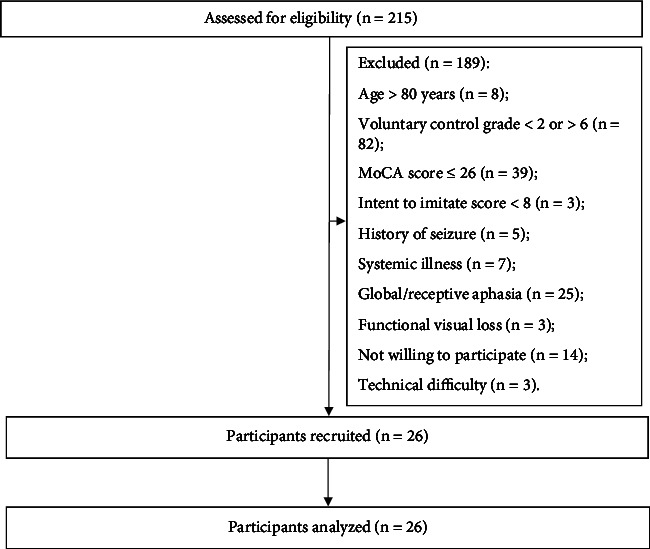
Flow of participants in the study.

A total number of 215 stroke survivors were screened for the study. Out of 215 patients, 189 did not meet the eligibility criteria. Twenty-six poststroke subjects (21 males, mean ± SD age of 53.4 ± 15.1 years, and five females, mean ± SD age of 61.8 ± 7.5 years) were finally recruited and analyzed in this study (Figure 4). All the included participants completed the experiment. No adverse effects were reported during and after the procedure. The study participants' demographic details are displayed in Table 1.

As can be seen from Table 1, participants predominantly consisted of ischemic stroke with an FM-UE score (mean ± SD) of 36.96 ± 17.19. No difference was seen in the side of the stroke involved. The median poststroke duration was 13.0 (6.50, 180.5) days. The mean ± SD score for BMRS, MOCA score, and intent to imitate score are given in Table 1.

### 3.2. Results of EEG Analysis

As depicted in Table 2, the results showed a statistically significant difference in mu suppression in C3 (*p* = <0.001) and C4 (*p* = <0.001) electrodes between the whole and part (proximal and distal components) tasks during AOI of the UL reach-to-grasp task. The post hoc analysis revealed significant suppression of mu rhythm during AOI of the whole task compared to the proximal and distal movements (part task) in both C3 and C4 electrodes (C3 (whole vs. proximal): observation (*p* = 0.013), imitation (*p* = 0.029); C3 (whole vs. distal): observation (0.009), imitation (*p* = 0.007); C4 (whole vs. proximal): observation (*p* = 0.024), imitation (*p* = 0.031); C4 (whole vs. distal): observation (0.014), imitation (*p* = 0.043) (Table 2). However, we could not find any significant difference between part components of the task (i.e., between proximal and distal components of the task).

### 3.3. Results of EMG Analysis

Friedman's ANOVA showed a statistically significant difference in %MVC amplitude in anterior deltoid (*p* = 0.002), supraspinatus (*p* = <0.001), triceps brachii (*p* = 0.002), brachioradialis (*p* = 0.006), and ECR (*p* = <0.001) muscles across the whole, proximal, and distal tasks during AOI of the UL reach-to-grasp task. The post hoc revealed a significant increase in muscle activity during imitation of the whole task compared to the part task (proximal and distal components of the task) in all the muscles except the biceps brachii (Table 3). Comparison of muscle activity between imitation of proximal and distal components of the task shows that AOI of proximal movement video leads to significant activation of proximal muscles, i.e., deltoid (*p* = 0.022), supraspinatus (*p* = 0.041), and triceps (*p* = 0.041), whereas AOI of distal movement video leads to distal muscle activation, i.e., brachioradialis (*p* = 0.015) and ECR (*p* = 0.001) (Table 3).

## 4. Discussion

This study examines the influence of AOI of the component of the part task compared to the whole task on muscle activity and functional connectivity following stroke. Both the EEG and EMG results are in favor of the whole task compared to the part task in terms of better brain activity and muscle activity. This could be because the observation of complex movements that require a higher level of motor control–like manipulation or reaching to grasp with the entire arm needs a higher level of sensory-motor coding, thereby leading to an increased activity of MNS [37]. The increased activity of MNS would have led to the activation of UL muscles due to the anatomical connections between the motor cortex and MNS [38, 39]. The previous studies have found that there are strong connections and modulatory influences between the MNS (specifically premotor area F5) and primary motor cortex [39, 40]. In our study, the correlation analysis between EEG and EMG signals revealed a statistically significant moderate positive correlation for supraspinatus (*r* = 0.406, *p* = 0.039) and triceps brachii (*r* = 0.477, *p* = 0.014) muscles during imitation of the whole task and a weak to no correlation during imitation of the part tasks. Therefore, we assume that the MNS activation in the cortex would have translated into the peripheral muscles during the AOI of the whole task. However, the results of the correlation analysis between the EEG and EMG signals should be interpreted cautiously due to the small sample size inadequate for correlation analysis [41]. We recommend that future studies should assess and report the correlation or association between MNS activity and peripheral muscle activity. Our EEG results are in line with a previous study conducted in normal healthy individuals, which concluded that observing the entire reach-to-grasp movement was more effective at activating MNS than only observing the proximal or distal parts of the reach-to-grasp movement [23].

The EMG results showed increased muscle activity with AOI of the whole task in all the muscles, especially triceps brachii and ECR which are considered as the important muscles required for the performance of reach-to-grasp task. The reduced muscle activity in the biceps brachii during the whole task could be attributed to the presence of spasticity in the elbow flexors [42]. Another important finding from our study is that AOI of the proximal component of the task elicited increased muscle activity in the deltoid, supraspinatus, and triceps brachii (proximal muscles) whereas AOI of the distal component of the task elicited increased muscle activity in brachioradialis and ECR (distal muscles). This may result from the muscle-specific activation of the primary motor cortex during mere observation of movements, thus leading to increased activity in the muscles participating in the observed motion exhibiting a higher degree of modulation than the muscles that are not [43]. The reason for the better functional connectivity and increased muscle activity in proximal muscles during imitation of proximal components of the task could be explained by the hierarchical activation of the proximal musculature first in order to drive the movement in space. Observation of the distal component of the task appears to elicit better functional connectivity and muscle activity in distal muscles, which could be attributed to the activation of the hand area that has a larger representation in the brain [44]. However, further research is warranted to ascertain this assumption.

There were a few limitations present in this study. Firstly, the distraction of the patients could not be completely eliminated due to the lengthy procedure even though tests were carried out in a controlled environment. However, the data retrieved were technically sound and eligible for analysis. Secondly, for all the participants, the videos consist of an actor performing the task with the right hand. For people with left-side involvement, we have not shown a separate video in which the actors complete the task with the left hand. Third, interference of EMG on EEG could not be avoided due to feasibility issues. Apart from this, we have not analyzed the other channels of EEG apart from the central C3 and C4 electrodes, and therefore, we could have missed analyzing the MNS present in the other important areas.

## 5. Conclusion

From the EMG and EEG results, we conclude that the whole task should be given emphasis while framing the AOI treatment module to improve the reach-to-grasp task in people with stroke. There seems to be a task observation–specific activation of muscles following AOI of proximal or distal tasks. Applying AOI of daily activities to current stroke rehabilitation programs should ameliorate the therapeutic benefits.

## Figures and Tables

**Table 1:** Demographic characteristics of study participants (*n* = 26).

**Variables**	**Descriptive statistics**
Age (years)	55.00 ± 14.49^a^
Gender (male:female)	21 (80%):5 (20%)^b^
Side of hemiparesis (right:left)	13 (50%):13 (50%)^b^
Type of stroke (ischemic:hemorrhagic)	19 (73%):7 (27%)^b^
Poststroke duration (days)	13.0 (6.50, 180.5)^c^
FM-UE score	
Motor	36.96 ± 17.194^a^
Sensory	12.00^a^
BMRS	
Upper limb	3.72 ± 1.17^a^
Hand	3.84 ± 1.28^a^
MOCA score	29.64 ± .907^a^
MAS score—elbow flexors	
Grade 0	10 (38.4%)^b^
Grade 1	11 (42.3%)^b^
Grade 1+	2 (7.6%)^b^
Grade 2	2 (7.6%)^b^
Grade 3	1 (3.8%)^b^
Wrist flexors	
Grade 0	21 (80.7%)^b^
Grade 1	1 (3.8%)^b^
Grade 2	4 (15.3%)^b^

^a^Mean ± standard deviation.

^b^Number (percentage).

^c^Median (Q1, Q3).

Abbreviations: BMRS = Brunnstrom motor recovery stage; FM-UE = Fugl-Meyer–Upper Extremity; MAS = modified Ashworth scale; MOCA = Montreal cognitive assessment.

**Table 2:** Comparison of mu suppression score (*μ*V) across test conditions (observation and imitation of the whole task and proximal and distal tasks) in the C3 and C4 areas (*n* = 26).

**EEG electrode placement areas**	**Friedman's ANOVA**	**AOI task**	**Movement conditions**			**Median difference (Q1, Q3)**	**p** ** value**
			**Whole movement median (Q1, Q3)**	**Proximal movement median (Q1, Q3)**	**Distal movement median (Q1, Q3)**		
C3	< 0.001^∗^	Observation	1.24 (0.46, 2.23)	2.56 (1.11, 4.72)		1.61	0.013^∗^
			1.24 (0.46, 2.23)		2.39 (0.97, 5.26)	1.00	0.009^∗^
				2.56 (1.11, 4.72)	2.39 (0.97, 5.26)	0.25	0.582
		Imitation	1.82 (0.88, 2.76)	3.23 (2.00, 5.15)		1.54	0.029^∗^
			1.82 (0.88, 2.76)		3.91 (1.90, 5.20)	1.55	0.007^∗^
				3.23 (2.00, 5.15)	3.91 (1.90, 5.20)	0.17	0.565

C4	< 0.001^∗^	Observation	1.47 (0.35, 2.83)	2.74 (1.84, 3.80)		0.89	0.024^∗^
			1.47 (0.35, 2.83)		2.40 (0.97, 4.67)	1.18	0.014^∗^
				2.59 (1.01, 3.49)	2.40 (0.97, 4.67)	0.24	0.227
		Imitation	1.72 (0.68, 2.63)	3.56 (2.31, 5.10)		1.47	0.031^∗^
			1.72 (0.68, 2.63)		3.82 (1.66, 5.64)	1.60	0.043^∗^
				3.12 (1.55, 4.51)	3.82 (1.66, 5.64)	0.19	0.367

Abbreviations: ANOVA, analysis of variance; AOI, action observation and imitation; EEG, electroencephalography; Q1, quartile 1; Q3, quartile 3.

^∗^ *p* ≤ 0.05.

**Table 3:** Comparison of normalized electromyographic amplitudes (%MVC) between test conditions (imitation of the whole task and proximal and distal tasks) (*n* = 26).

**EMG electrode placement muscles**	**Friedman's ANOVA**	**Movement conditions**			**p** ** value**
		**Imitation of whole movement (Q1, Q3)**	**Imitation of proximal movement (Q1, Q3)**	**Imitation of distal movement (Q1, Q3)**	
Deltoid	0.002^∗^	52.61 (42.64, 58.70)	43.67 (36.41, 51.37)		0.008^∗^
		52.61 (42.64, 58.70)		33.44 (25.78, 44.86)	< 0.001^∗^
			43.67 (36.41, 51.37)	33.44 (25.78, 44.86)	0.022^∗^

Supraspinatus	< 0.001^∗^	69.00 (59.66, 76.64)	56.76 (46.52, 68.74)		0.031^∗^
		69.00 (59.66, 76.64)		48.54 (41.7, 54.8)	< 0.001^∗^
			56.76 (46.52, 68.74)	48.54 (41.7, 54.8)	0.041^∗^

Biceps brachii	0.446	46.81 (35.83, 57.00)	50.01 (37.81, 58.20)		0.940
		46.81 (35.83, 57.00)		41.89 (32.3, 52.6)	0.394
			50.01 (37.81, 58.20)	41.89 (32.3, 52.6)	0.247

Triceps brachii	0.002^∗^	74.26 (67.89, 81.52)	67.80 (53.61, 73.55)		0.006^∗^
		74.26 (67.89, 81.52)		59.66 (47.4, 69.1)	< 0.001^∗^
			67.80 (53.61, 73.55)	59.66 (47.4, 69.1)	0.041^∗^

Brachioradialis	< 0.001^∗^	50.66 (41.9, 55.9)	32.30 (18.7, 46.3)		< 0.001^∗^
		50.66 (41.9, 55.9)		43.55 (33.2, 46.9)	0.041^∗^
			32.30 (18.7, 46.3)	43.55 (33.2, 46.9)	0.015^∗^

Extensor carpi radialis	< 0.001^∗^	70.37 (60.3, 81.2)	26.40 (16.5, 43.2)		< 0.001^∗^
		70.37 (60.3, 81.2)		44.08 (37.09, 53.82)	< 0.001^∗^
			26.40 (16.5, 43.2)	44.08 (37.09, 53.82)	0.001^∗^

Abbreviations: %MVC, percentage maximum voluntary contraction; ANOVA, analysis of variance; EMG, electromyography; Q1, quartile 1; Q3, quartile 3.

^∗^ *p* ≤ 0.05.

## Data Availability

All data underlying the results is available as part of the article, and no additional source data is required.
